# Tracking Private WhatsApp Discourse About COVID-19 in Singapore: Longitudinal Infodemiology Study

**DOI:** 10.2196/34218

**Published:** 2021-12-23

**Authors:** Edina YQ Tan, Russell RE Wee, Young Ern Saw, Kylie JQ Heng, Joseph WE Chin, Eddie MW Tong, Jean CJ Liu

**Affiliations:** 1 Division of Social Sciences Yale-NUS College Singapore Singapore; 2 Centre for Sleep and Cognition Yong Loo Lin School of Medicine Singapore Singapore; 3 Department of Psychology National University of Singapore Singapore Singapore; 4 Neuroscience and Behavioral Disorders Program Duke-NUS Medical School Singapore Singapore; 5 Centre for Trusted Internet and Community National University of Singapore Singapore Singapore

**Keywords:** social media, WhatsApp, infodemiology, misinformation, COVID-19, tracking, surveillance, app, longitudinal, Singapore, characteristic, usage, pattern, well-being, communication, risk

## Abstract

**Background:**

Worldwide, social media traffic increased following the onset of the COVID-19 pandemic. Although the spread of COVID-19 content has been described for several social media platforms (eg, Twitter and Facebook), little is known about how such content is spread via private messaging platforms, such as WhatsApp (WhatsApp LLC).

**Objective:**

In this study, we documented (1) how WhatsApp is used to transmit COVID-19 content, (2) the characteristics of WhatsApp users based on their usage patterns, and (3) how usage patterns link to COVID-19 concerns.

**Methods:**

We used the experience sampling method to track day-to-day WhatsApp usage during the COVID-19 pandemic. For 1 week, participants reported each day the extent to which they had received, forwarded, or discussed COVID-19 content. The final data set comprised 924 data points, which were collected from 151 participants.

**Results:**

During the weeklong monitoring process, most participants (143/151, 94.7%) reported at least 1 COVID-19–related use of WhatsApp. When a taxonomy was generated based on usage patterns, around 1 in 10 participants (21/151, 13.9%) were found to have received and shared a high volume of forwarded COVID-19 content, akin to super-spreaders identified on other social media platforms. Finally, those who engaged with more COVID-19 content in their personal chats were more likely to report having COVID-19–related thoughts throughout the day.

**Conclusions:**

Our findings provide a rare window into discourse on private messaging platforms. Such data can be used to inform risk communication strategies during the pandemic.

**Trial Registration:**

ClinicalTrials.gov NCT04367363; https://clinicaltrials.gov/ct2/show/NCT04367363

## Introduction

WhatsApp (WhatsApp LLC) is the most commonly used messaging app worldwide; it has 1.5 billion users across 180 countries [1]. On account of its large user base and near-instant message transmission capabilities, the platform has played a critical role in risk communication during the COVID-19 pandemic.

WhatsApp has been co-opted by government agencies and the World Health Organization to disseminate official COVID-19 updates [2]. However, while this showcases the platform’s ability to reach a large sector of the population, this feature has also made it a vessel for misinformation. For example, at the beginning of the pandemic, WhatsApp noted a 40% surge in usage [3]. This was paired with a high volume of message forwarding activity that was widely believed to support misinformation. As a result, the platform restricted the number of individuals to whom a message could be forwarded simultaneously [4,5].

Despite these restrictions, a survey in India found that 1 in 2 participants had received COVID-19 misinformation through WhatsApp or Facebook [6]. Likewise, WhatsApp was identified by Hong Kong residents as the foremost source for COVID-19–related rumors [7]. As misinformation can jeopardize public health strategies, these findings underscore the need for infodemiological studies that document how COVID-19 content spreads through WhatsApp.

To date however, the bulk of infodemiology studies have focused on social media platforms in which content is publicly accessible (eg, Twitter and Facebook) [8,9]. In contrast, research on WhatsApp has proven to be elusive because of the platform’s private nature; its end-to-end encryption software ensures that only senders and recipients have access to messages sent through the platform. Nonetheless, WhatsApp research remains a priority; aside from its popularity and role in disseminating crisis-related misinformation [10,11], insights from public posts are also unlikely to generalize to WhatsApp’s private messages [12]. It thus remains unclear as to who sends COVID-19–related messages, who receives such messages, and what manner such messages are sent.

To address these gaps in the literature, we designed a study to (1) describe the base rate of COVID-19 content dissemination, (2) understand WhatsApp users, and (3) examine correlates of usage patterns. Specifically, we used the experience sampling method to track WhatsApp usage amid everyday routines across 1 week [13,14]. We asked participants to report each day their frequency of receiving, forwarding, or discussing COVID-19–related content. Through this method, we generated a taxonomy of participants based on their usage patterns and examined whether day-to-day variations in WhatsApp usage predicted COVID-19–related concerns.

## Methods

### Recruitment

From March 17 to May 7, 2020, participants were recruited from the general community via advertisements placed in Facebook and WhatsApp community groups (eg, residential groups, workplace groups, and university groups), posts on popular web-based forums, and paid Facebook advertisements targeting Singapore-based users. All study activities took place on the web-based survey platform Qualtrics (Qualtrics International Inc), and participants were reimbursed with SGD $5 (US $3.65) upon study completion. The study protocol was approved by the Yale-NUS (National University of Singapore) College Ethics Review Committee (protocol record: 2020-CERC-001) and was preregistered at ClinicalTrials.gov (trial number: NCT04367363).

### Participants

The participants were 151 adults who met the following inclusion criteria: (1) aged 21 years or older, (2) had lived in Singapore for at least 2 years, and (3) had a WhatsApp account.

### Measures

**Figure 1:**
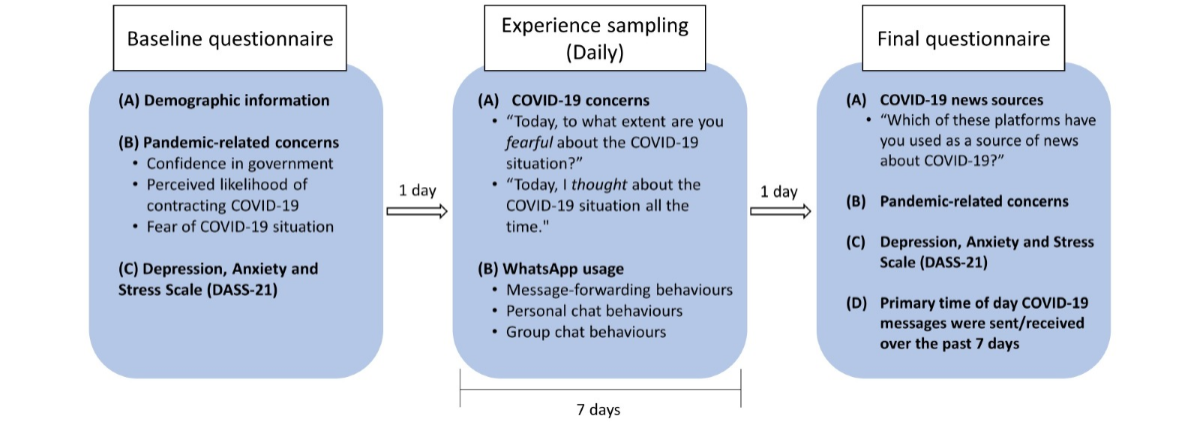
Schematic of study procedures. All participants completed a baseline questionnaire. This was followed by 7 days of experience sampling, during which participants addressed questions about COVID-19 concerns and WhatsApp usage daily. Participants completed a final questionnaire 1 day after the experience sampling procedure ended. DASS-21: 21-item Depression, Anxiety and Stress Scale.

Following the provision of informed consent, participants completed (1) a baseline questionnaire, (2) experience sampling responses daily for 7 days, and (3) a final questionnaire (Figure 1).

#### Experience Sampling

As the primary form of data collection, we used the experience sampling method to capture COVID-19 chatter on WhatsApp. Through this method, we collected 924 data points across 151 participants (compliance rate: 924/1057, 87.4%).

For 7 days, participants accessed a web-based survey each evening (at 9:30 PM) to report their WhatsApp usage for the day. Participants indicated whether they had forwarded messages related to COVID-19 (“yes” or “no”). We focused on message forwarding as a proxy indicator for high-risk content, since (1) large Twitter studies have observed that misinformation is more likely to be shared than posts that are true [15] and (2) WhatsApp developers had previously linked forwarded messages to misinformation [4,5]. If participants had forwarded COVID-19 content, they were then asked about (1) the number of unique COVID-19 messages they had forwarded and (2) the number of unique groups and individuals to which they had forwarded messages.

Participants were also asked about their personal chats (ie, their one-to-one chats on WhatsApp). They indicated whether COVID-19 messages had been forwarded to them in personal chats (“yes” or “no”). If so, they were asked about (1) the number of unique messages they had received and (2) the number of different people from which they had received messages. Thereafter, participants recounted whether they had discussed COVID-19 in conversations where either they or the other party generated messages related to COVID-19 (“yes” or “no”). If so, they were asked about how many unique chats were involved.

Finally, for group chats, participants were asked if COVID-19 had been mentioned in any of their WhatsApp groups by at least 1 other person (not including themselves; “yes” or “no”). This could have occurred either through others forwarding messages or through others generating their own comments. Affirmative responses were followed with a question on how many WhatsApp groups had done so.

Aside from WhatsApp metrics, participants also reported their COVID-19 concerns for the day; they were asked about (1) how afraid they felt about the COVID-19 situation (4-point scale: 1=“Not scared at all”; 4=“Very scared”) and (2) whether they thought about the COVID-19 situation all the time (5-point scale: 1=“Not at all true”; 5=“Very true”).

#### Baseline and Final Questionnaires

To characterize the participants, we included baseline and final questionnaires in which participants reported demographics (age, gender, religion, ethnicity, marital status, education, house type, household size, citizenship, country of birth, and number of years in Singapore), the time of day when they read and sent COVID-19 messages on WhatsApp (mostly in the morning, afternoon, evening, or late night or throughout the day), and sources through which they obtained COVID-19 news (eg, printed newspapers, radio, WhatsApp, and YouTube). Additionally, participants completed the 21-item Depression, Anxiety and Stress Scale (DASS-21) [16] to evaluate their mental health during the pandemic. Participants were also asked about their responses to the pandemic [2,17], that is, (1) how confident they were that the government could control the nationwide spread of COVID-19 (1=“Not confident at all; 4=“Very confident”), (2) their perceptions on how likely that they or someone in their immediate household would contract COVID-19 (1=“Not at all likely”; 4=“Very likely”), and (3) how fearful they were about the situation in the country (1=“Not scared at all”; 4=“Very scared”).

### Statistical Analysis

**Figure 2:**
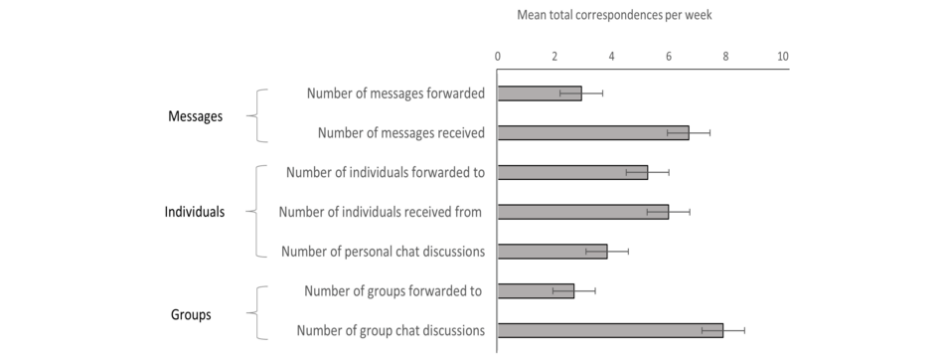
Distribution of COVID-19–related behaviors on WhatsApp. In a weeklong experience sampling procedure, participants reported the extent to which they engaged in COVID-19–related behaviors on WhatsApp (either by forwarding or receiving messages or in conversations). Horizontal bars represent the total amount of each activity captured (averaged across all participants). Horizontal lines represent the 95% CIs for the means.

First, we summarized the data as counts with percentages or as means with SDs, focusing on the following seven quantitative WhatsApp usage variables (Figure 2): the number of (1) COVID-19 messages that participants forwarded, (2) groups to whom messages were forwarded, (3) individuals to whom messages were forwarded, (4) forwarded messages received, (5) individuals from whom messages were received, (6) personal chats involving COVID-19–related conversations, and (7) group chats discussing COVID-19. Multimedia Appendix 1 shows the pattern of correlations across these variables.

Second, to understand WhatsApp user profiles, we performed a latent profile analysis to create a taxonomy of participants based on their WhatsApp usage (R package *mclust* [18]). Latent profile analysis is a bottom-up statistical clustering method for defining classes of people based on common characteristics. By using all observations of a continuous dependent variable, classes are created such that within each class, indicator variables are statistically uncorrelated [19]. We thus used this technique to cluster participants based on their responses to the seven WhatsApp usage variables; values were obtained by aggregating the reported frequency of each variable over the week. To uncover clusters, we used Gaussian mixture models and assigned participants to clusters by using Bayesian probabilities. The final number of clusters was determined by using the Bayesian information criterion, the integrated completed likelihood criterion, and a bootstrap likelihood test.

Finally, we examined whether day-to-day variations in COVID-19 WhatsApp chatter could be tracked based on variations in COVID-19 concerns. We quantified COVID-19 chatter on personal and group chats, so that such chatter could be used as predictors. For personal chats, the following variables were summed for each day and for each participant: the number of (1) individuals to whom COVID-19 messages were forwarded, (2) individuals from whom forwarded messages were received, and (3) personal conversations discussing COVID-19. For group chats, the following variables were summed: the number of (1) groups participants to whom COVID-19 messages were forwarded and (2) groups where COVID-19 messages were mentioned. Scores were grand-mean centered by subtracting the mean number of chats across subjects and time points from each score (number of chats: mean 2.47 and mean 1.29, respectively). In addition, we created between- and within-subject versions of each predictor [20]. The final analyses involved linear mixed-effects models for each outcome measure (fear of and thoughts about COVID-19). The following were entered as fixed effects: time (centered such that 0 referred to the middle of the week), daily personal chats (between subjects), daily personal chats (within subjects), daily group chats (between subjects), and daily group chats (within subjects). Random intercepts accounted for correlated data resulting from repeated measures.

Across all analyses, the type 1 decision-wise error rate was controlled at an α of .05. All statistical analyses were conducted in R 3.5.0 (R Foundation for Statistical Computing) and SPSS 25 (IBM Corporation).

## Results

### Baseline Participant Characteristics

As shown in Table 1, 68.9% (104/151) of participants were female, and their mean age was 36.35 (SD 14.7) years. Participants were predominantly of Asian ethnicity (Chinese: 140/151, 92.7%) and had at least a postsecondary education (133/151, 88.1%). Further, 39.7% (60/151) of participants were married, and the majority (105/151, 69.5%) belonged to households of at least 4 members.

**Table 1:** Participant characteristics as a function of COVID-19 WhatsApp usage patterns.

Characteristic			Chronic users (n=21)		Receiving users (n=47)		Discursive users (n=46)		Minimal users (n=37)		All participants (N=151)
Age (years), mean (SD)			44.1 (14.5)		41.0 (15.5)		29.7 (10.7)		34.4 (14.5)		36.35 (14.70)
**Gender, n (%)**											
	Female	13 (62)		34 (72)		29 (63)		28 (76)		104 (69)	
	Male	8 (38)		13 (28)		17 (37)		9 (24)		47 (31)	
**Ethnicity** **, n (%)**											
	Chinese	20 (95)		42 (89)		42 (91)		36 (97)		140 (93)	
	Indian	0 (0)		2 (5)		3 (7)		0 (0)		5 (3)	
	Malay	0 (0)		2 (5)		1 (2)		0 (0)		3 (2)	
	Other	1 (5)		1 (1)		0 (0)		1 (3)		3 (2)	
**Religion, n (%)**											
	Christianity (Protestant)	8 (38)		17 (36)		16 (35)		13 (35)		54 (36)	
	No religion	3 (14)		14 (30)		11 (24)		10 (27)		38 (25)	
	Buddhism	4 (19)		9 (19)		8 (18)		11 (30)		32 (21)	
	Roman Catholicism	4 (19)		4 (9)		6 (13)		2 (5)		16 (11)	
	Taoism or Chinese traditional beliefs	1 (5)		0 (0)		2 (4)		1 (3)		4 (3)	
	Islam	1 (5)		3 (6)		1 (2)		0 (0)		5 (3)	
	Hinduism	0 (0)		0 (0)		2 (4)		0 (0)		2 (1)	
**Marital status** **, n (%)**											
	Married	13 (62)		24 (51)		8 (17)		15 (41)		60 (40)	
	Single	6 (28)		15 (32)		25 (55)		12 (32)		58 (38)	
	Dating	1 (5)		7 (15)		12 (26)		9 (24)		29 (19)	
	Widowed, separated, or divorced	1 (5)		1 (2)		0 (0)		1 (3)		3 (2)	
	Did not answer	0 (0)		0 (0)		1 (2)		0 (0)		1 (1)	
**Educational level, n (%)**											
	O level	1 (5)		4 (9)		1 (2)		6 (16)		12 (8)	
	Junior college	2 (10)		5 (10)		9 (19)		9 (24)		25 (17)	
	Institute of Technical Education	1 (5)		1 (2)		1 (2)		0 (0)		3 (2)	
	Polytechnic or diploma	2 (10)		13 (28)		7 (15)		4 (11)		26 (17)	
	University (undergraduate)	11 (51)		21 (45)		21 (46)		16 (43)		69 (46)	
	University (postgraduate)	4 (19)		1 (2)		3 (7)		2 (6)		10 (7)	
	Other	0 (0)		2 (4)		3 (7)		0 (0)		5 (3)	
	Did not answer	0 (0)		0 (0)		1 (2)		0 (0)		1 (1)	
**House type, n (%)**											
	HDB^a^ flat (1-2 rooms)	0 (0)		0 (0)		0 (0)		1 (3)		1 (1)	
	HDB flat (3 rooms)	0 (0)		2 (4)		2 (4)		2 (5)		6 (4)	
	HDB flat (4 rooms)	2 (10)		9 (19)		10 (22)		10 (27)		31 (21)	
	HDB flat (5 rooms)	3 (14)		19 (40)		14 (31)		11 (30)		47 (31)	
	Condominium	12 (57)		12 (26)		11 (24)		10 (27)		45 (30)	
	Landed property	4 (19)		4 (9)		7 (15)		2 (5)		17 (11)	
	Did not answer	0 (0)		1 (2)		2 (4)		1 (3)		4 (3)	
**Household size (number of members), n (%)**											
	1	2 (10)		1 (2)		3 (7)		0 (0)		6 (4)	
	2	0 (0)		5 (11)		3 (7)		3 (8)		11 (7)	
	3	7 (33)		8 (17)		5 (11)		8 (22)		28 (19)	
	4	7 (33)		18 (38)		21 (45)		15 (40)		61 (40)	
	≥5	5 (24)		15 (32)		13 (28)		11 (30)		44 (29)	
	Did not answer	0 (0)		0 (0)		1 (2)		0 (0)		1 (1)	
**Citizenship, n (%)**											
	Singapore	18 (86)		46 (98)		42 (91)		36 (97)		142 (94)	
	Other	3 (14)		1 (2)		4 (9)		1 (3)		9 (6)	
**Country of birth, n (%)**											
	Singapore	17 (81)		45 (96)		38 (83)		33 (89)		133 (88)	
	Other	4 (19)		2 (4)		8 (17)		4 (11)		18 (12)	
Number of years in Singapore, mean (SD)			39.67 (15.22)		39.60 (16.69)		26.43 (10.83)		31.65 (14.47)		33.65 (15.32)
**21-item Depression, Anxiety and Stress Scale scores, mean (SD)**											
	Stress	9.52 (7.12)		8.61 (7.08)		9.56 (10.13)		10.81 (8.72)		9.57 (8.47)	
	Anxiety	4.38 (5.28)		5.13 (5.44)		5.33 (6.59)		5.89 (7.71)		5.28 (6.36)	
	Depression	8.10 (6.52)		7.22 (6.86)		9.47 (9.73)		10.76 (9.54)		8.90 (8.50)	
**Pandemic-related concerns (score), mean (SD)**											
	Fear of COVID-19 situation	2.29 (0.46)		2.53 (0.65)		2.22 (0.74)		2.27 (0.69)		2.34 (0.67)	
	Confidence in government	3.33 (0.58)		3.23 (0.63)		3.29 (0.66)		3.24 (0.72)		3.27 (0.65)	
	Perceived likelihood of contracting COVID-19	2.71 (0.64)		2.74 (0.53)		2.78 (0.56)		2.76 (0.60)		2.75 (0.57)	

^a^HDB: housing and development.

### Base Rate of COVID-19 WhatsApp Usage

**Figure 3:**
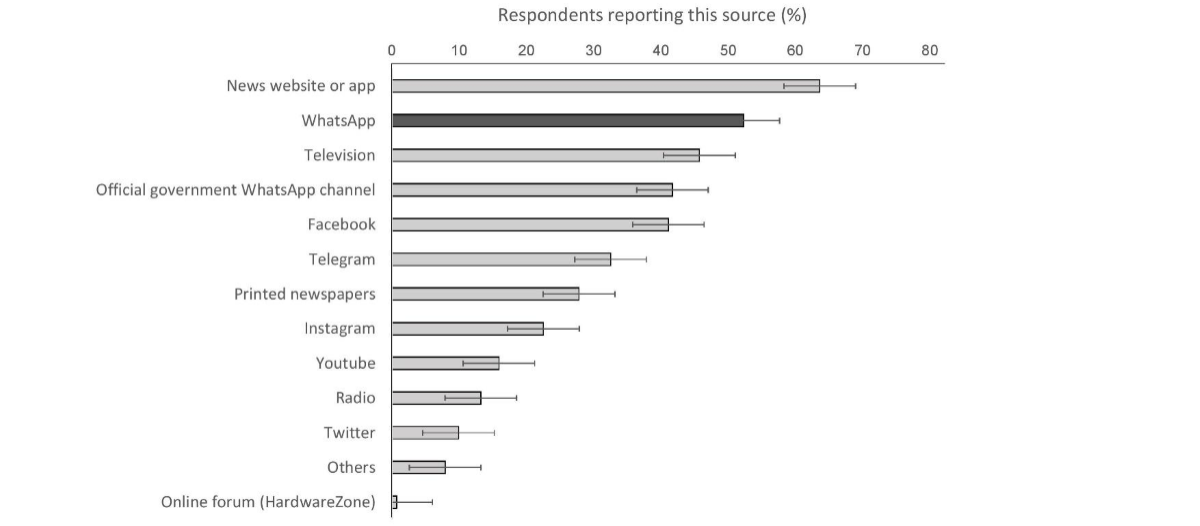
Sources of COVID-19 news. In a questionnaire, participants self-reported the sources from which they received COVID-19 news.

Participants’ self-reports revealed that WhatsApp was the second most common source for COVID-19 news after news websites or apps (Figure 3). By quantifying this through 1 week of experience sampling, we found that nearly all participants (143/151, 94.7%; 95% CI 90-98%) reported at least 1 COVID-19 related use of WhatsApp. Namely, around 1 in 2 participants (79/151, 52.3%; 95% CI 44%-60%) forwarded at least 1 COVID-19 message (to either individuals or groups), 78.1% (118/151; 95% CI 71%-84%) received at least 1 forwarded message in personal chats, 66.2% (100/151; 95% CI 58%-74%) engaged in personal chat conversations about COVID-19, and 88.1% (133/151; 95% CI 82%-93%) had been in groups where COVID-19 was mentioned.

Figure 2 shows the extent to which participants engaged in each of these activities. On average, participants (1) received 2.3 times more messages than they forwarded and (2) were more likely to forward messages to individuals than to groups (average of 5.3 messages per week vs 2.7 messages per week, respectively). Beyond passive engagement, participants also took part in an average of 3.8 one-to-one conversations about COVID-19 during the week; however, these interactions occurred less frequently than the sending or receiving of forwarded messages in group chats.

### Characterizing Participants Based on COVID-19 WhatsApp Usage

#### Latent Profile Analysis: Generating a Taxonomy of WhatsApp Usage

**Figure 4:**
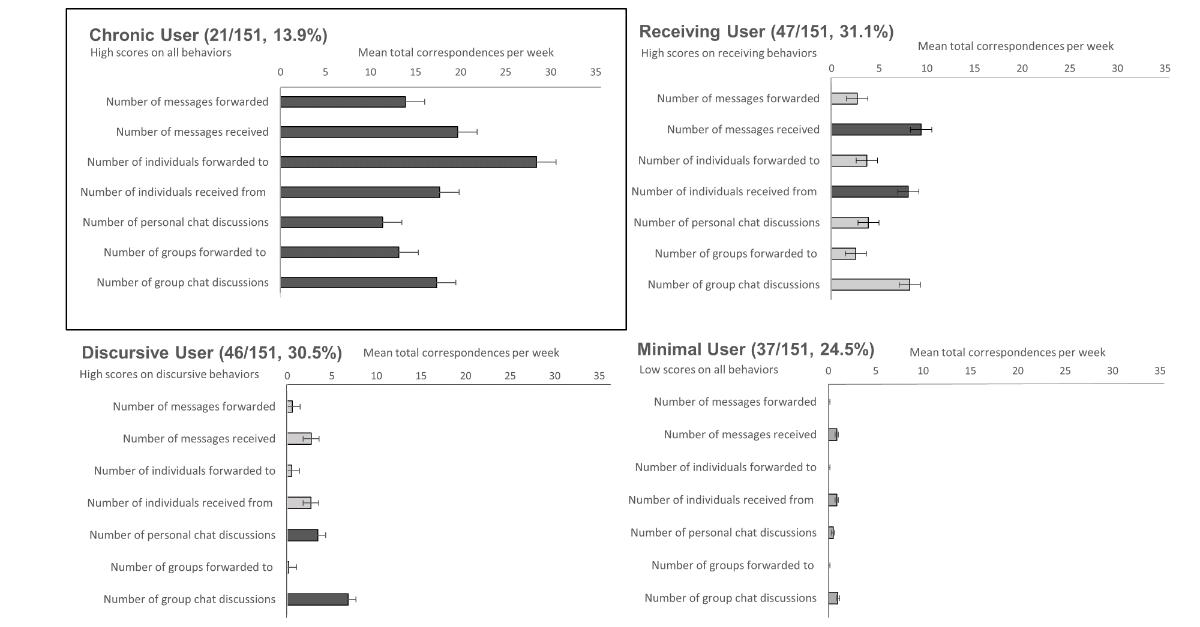
Taxonomy of COVID-19–related WhatsApp usage. By using latent profile analysis, we classified participants based on how they had used WhatsApp to engage with COVID-19 content during 1 week of monitoring. The figure depicts the WhatsApp usage activities of chronic users (top left), receiving users (top right), discursive users (bottom left), and minimal users (bottom right). Horizontal lines represent the 95% CIs for the means.

Although most participants (143/151, 94.7%) received and shared COVID-19 content on WhatsApp, there were individual differences in usage patterns (Figure 4). Correspondingly, we conducted a latent profile analysis to understand how usage patterns clustered.

A 4-cluster solution yielded the lowest absolute Bayesian information criterion values (Multimedia Appendix 2), resulting in the following taxonomy (Figure 4). First, 13.9% (21/151) of participants were chronic users, who exhibited high levels of activity with regard to each of the WhatsApp usage variables. Correspondingly, this group of participants was responsible for receiving and transmitting a large volume of forwarded COVID-19 messages; they sent the messages both to individual contacts and to groups. Second, 31.1% (47/151) of participants were receiving users, who were distinguished by their receipt of multiple forwarded COVID-19 messages. Although this group discussed COVID-19 frequently in group chats, they rarely passed along the forwarded COVID-19 messages that they had received. A third group – discursive users (46/151, 30.5%) – had low exposure to forwarded COVID-19 messages and primarily engaged with COVID-19 content through personal and group chats. Finally, 24.5% (37/151) of participants were minimal users, who had low levels of engagement with COVID-19 content overall.

#### Understanding User Characteristics

As an exploratory analysis, we performed a classification tree analysis to predict WhatsApp user types based on demographics, COVID-19 concerns, depression and anxiety scores (DASS-21), and the time of day when participants used WhatsApp. We performed recursive partitioning (*rpart*)—a machine learning technique that allows multiple variables to be analyzed simultaneously and supports the modeling of complex, nonlinear relations among predictors [21]. To avoid overfitting, the final tree was pruned by selecting the tree size with the lowest cross-validation error, which, for our data set, was a tree size of 8.

**Figure 5:**
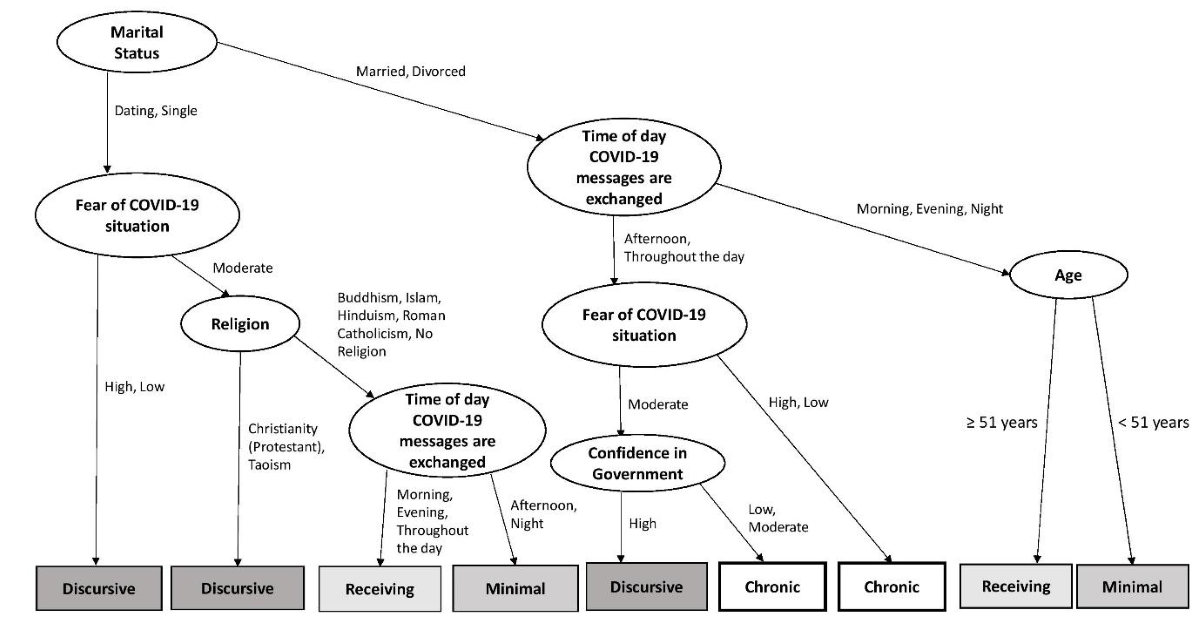
Classification tree analysis. Recursive partitioning was used to predict which of the four WhatsApp usage profiles (chronic, receiving, discursive, or minimal) participants belonged to based on baseline questionnaire measures (demographics; COVID-19 concerns; scores on the 21-item Depression, Anxiety and Stress Scale; and time of WhatsApp usage). The final tree model is presented as a flowchart; factors are chosen at each level to categorize the maximal number of participants. Marital status, the time of WhatsApp usage, and age emerged as the primary predictors (model classification accuracy: 64.2%; above the chance level of 25%).

As shown in Figure 5, chronic users were more likely to be married or divorced and more likely to send messages either throughout the day or in the afternoon. In terms of responses to the COVID-19 pandemic, chronic users either (1) had extreme fears of the COVID-19 situation (low or high levels of fear) or (2) had moderate fears paired with low confidence in the government’s response (low or moderate confidence in government).

Discursive users were more likely to be single or be dating and had either (1) extreme levels of COVID-19–related fears (either high or low levels of fear) or (2) moderate fear levels alongside Christian or Taoist affiliations. A subgroup of discursive users were, like chronic users, married or divorced and had moderate levels of COVID-19–related fears. However, they were distinguished from chronic users based on their high confidence in the government (chronic users had lower confidence in the government).

Finally, receiving, and minimal users had similar profiles. If they were single or were dating, both sets of users tended to have moderate levels of COVID-19–related fears, had a wide range of religious backgrounds, and were distinguished based on the time of day when they received COVID-19–related messages (receiving users: in the morning, in the evening, and throughout the day; minimal users: in the afternoon and at night). If they were married or divorced, both sets of users tended to send messages at only 1 time of the day (morning, evening, or night) and were distinguished based on age (receiving users: aged ≥51 years; minimal users: aged <51 years). Table 1 describes the demographic characteristics of the four user profiles.

#### Does WhatsApp Usage Relate to COVID-19 Concerns?

**Figure 6:**
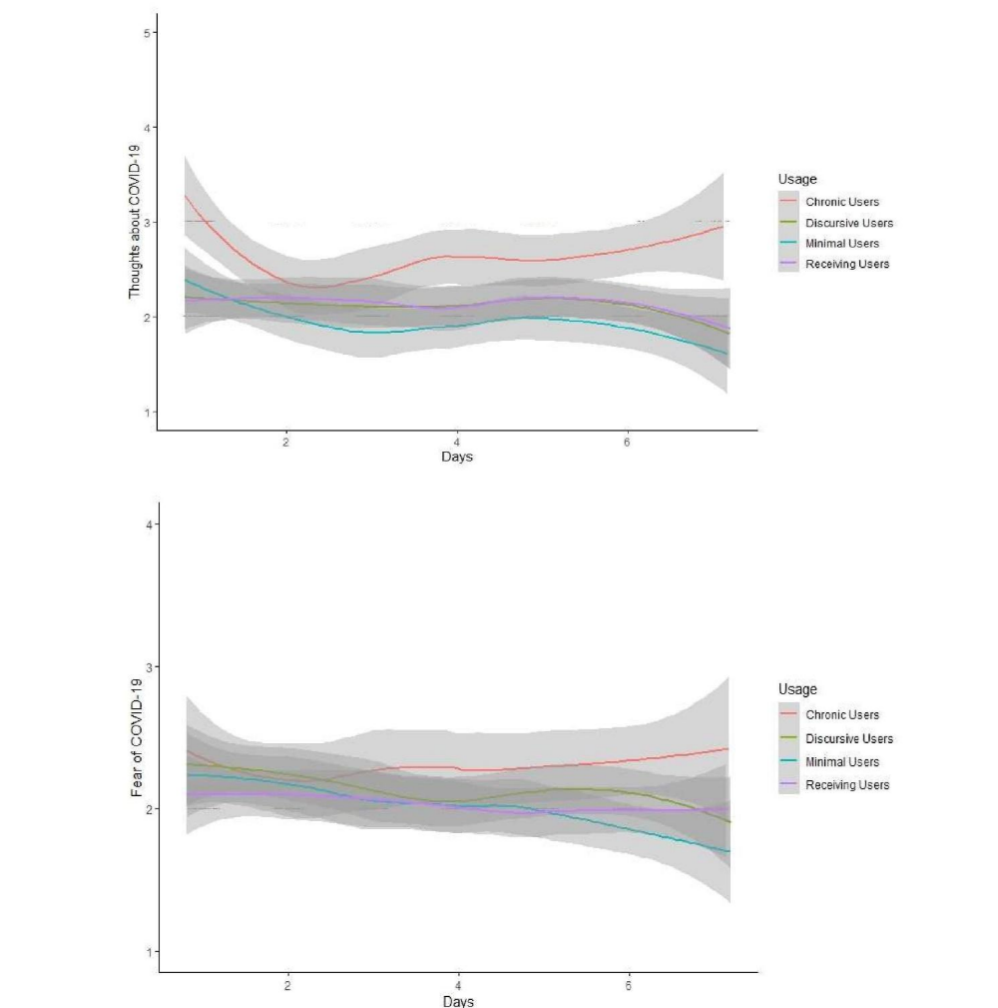
COVID-19–related thoughts and fears over 1 week. Day-to-day variations in COVID-19–related thought (top) and fear levels (bottom) as a function of WhatsApp user profiles. The shaded grey areas represent 95% CIs.

Finally, we conducted linear mixed-effects models to examine whether WhatsApp usage was related to COVID-19 concerns (Table 2). As shown in Figure 6, day-to-day COVID-19–related fears and thoughts fluctuated (fears: t_249.13_=−3.72; *P*<.001; thoughts: t_297.02_=−2.36; *P*=.02).

**Table 2:** Parameter estimates for the multi-level model of thoughts about COVID-19 (model 1) and the fear of COVID-19 (model 2) as a function of participants’ daily WhatsApp use (personal chats and group chats).

Model and effects				Estimate, β (SE; 95% CI)		*t* test^a^ *(df*) or *Z*		*P* value
**Model 1 outcome: thoughts about COVID-19**								
	**Fixed effects**							
		Intercept	2.18 (0.07; 2.05 to 2.31)		32.81 (135.68)		<.001	
		Time (centered)	−.03 (.01; −.05 to 0)		−2.36 (297.02)		.02	
		Daily personal chat usage (between subjects)	.04 (.02; 0 to .07)		2.36 (164.48)		.02	
		Daily personal chat usage (within subjects)	0 (.01; −.01 to .02)		0.42 (17.63)		.68	
		Daily group chat usage (between subjects)	.05 (.06; −.06 to .17)		0.89 (141.17)		.37	
		Daily group chat usage (within subjects)	0 (.03; −.06 to .05)		−0.08 (14.09)		.93	
	**Random effects**							
		Intercept (between subjects)	.56 (.08; .42 to .75)		6.89		<.001	
		Residual (within subjects)	.37 (.02; .33 to .43)		14.90		<.001	
		Autocorrelation (within subjects)	.24 (.05; .14 to .33)		4.97		<.001	
**Model 2 outcome: fear of COVID-19**								
	**Fixed effects**							
		Intercept	2.10 (0.06; 1.98 to 2.21)		36.37 (144.90)		<.001	
		Time (centered)	−.03 (.01; −.05 to −.02)		−3.72 (249.13)		<.001	
		Daily personal chat usage (between subjects)	.01 (.01; −.02 to .04)		0.85 (155.44)		.39	
		Daily personal chat usage (within subjects)	.01 (.01; −.01 to .02)		1.22 (24.97)		.24	
		Daily group chat usage (between subjects)	.02 (.05; −.07 to .12)		0.49 (128.59)		.62	
		Daily group chat usage (within subjects)	−.03 (.02; −.06 to .01)		−1.42 (28.88)		.17	
	**Random effects**							
		Intercept (between subjects)	.44 (.06; .34 to .58)		7.47		<.001	
		Residual (within subjects)	.21 (.01; .18 to .23)		13.83		<.001	
		Autocorrelation (within subjects)	.26 (.05; to .16 to .35)		5.16		<.001	

^a^The *t* test was 2-tailed.

For thoughts about COVID-19, there was a significant effect in WhatsApp personal chat usage at a between-subjects level (t_164.48_=2.36; *P*=.02), that is, participants who handled larger amounts of COVID-19 content in their personal chats reported having more thoughts about COVID-19 (relative to participants who handled smaller amounts of COVID-19 content). However, the corresponding effect for group chats was not significant (t_141.17_=0.89; *P*=.37). At the within-subjects level, neither day-to-day fluctuations in personal chat activities nor those in group chat activities significantly predicted thoughts about COVID-19 (smallest *P*=.68).

For COVID-19–related fears, we found no significant effects in any of the WhatsApp usage variables (smallest *P*=.17). For sensitivity analyses, we repeated both models and used group membership as a fixed factor in place of personal and group chat usage, and our primary conclusions did not change (Multimedia Appendix 3).

## Discussion

### Principal Findings

The ongoing pandemic has drawn attention to the role of social media in public health. Against this backdrop, we present the first infodemiological study to document the spread of COVID-19 content through WhatsApp. By tracking daily WhatsApp usage for 1 week, we found that (1) nearly every participant engaged in COVID-19 chatter and (2) participants were more likely to share or receive forwarded messages than to engage in conversations about COVID-19.

The volume of forwarded messages that we observed raises concern. On other social media platforms, forwarding behaviors have been linked to the spread of misinformation. For example, a study of 4.5 million Twitter posts found that misinformation was 70% more likely to be shared than posts that were true; correspondingly, any single retweet had a higher probability of containing false news rather than truthful news [15]. Although analogous research has not been conducted on WhatsApp, the app’s developers have likewise deemed forwarded messages as a high-risk source of misinformation [4,5]. 

With regard to the extent that forwarded messages carry misinformation [1,2], our latent profile analyses revealed that around 1 in 10 (21/151, 13.9%) participants were chronic users who received and shared a large volume of such messages. Notably, chronic users disseminated an average of 14 forwarded messages during the week, which is approximately 5 times the number of messages that were sent by all participants in this study. This is reminiscent of research on other social media platforms (eg, Twitter) where a small group of super sharers and super consumers are responsible for the bulk of shared misinformation [22]. Given the potential influence of this group, further research is needed to understand (1) the profile of chronic users, (2) the reasons why they forward messages, and (3) how their forwarding activities may influence outcomes during health crises.

Aside from chronic users, our study also found that around 1 in 3 (47/151, 31.1%) participants were receiving users who had high exposure to forwarded COVID-19 content. Receiving users tended to be older (in line with misinformation studies on Facebook [23]) but were otherwise moderate in terms of their profiles, that is, in terms of COVID-19–related fears or religion (they came from a diverse religious background). Although this group did not spread forwarded messages themselves, their high exposure may nonetheless leave them susceptible to false beliefs. Correspondingly, we urge researchers to conduct further research to understand how WhatsApp use among receiving users influences health behaviors.

Finally, we also found that WhatsApp users who discussed COVID-19 in their personal chats were more likely to think about COVID-19 throughout the day. As similar forms of rumination (involving frequent and persistent thoughts) have been linked to clinical depression [24], this finding may implicate COVID-19 chatter as a risk factor for poorer well-being [25]. Future studies should thus explore this possibility and the potential mechanisms involved.

### Implications

Taken together, our findings on WhatsApp message transmission have several implications for public health responses during a crisis. First, our taxonomy of user profiles provides a basis for targeted risk communication. Our findings suggest that public health agencies may need to reach out proactively to chronic and receiving users, who handle the bulk of forwarded COVID-19 content on WhatsApp. One possible intervention may be encouraging these users to subscribe to official WhatsApp channels for updates (eg, updates from the World Health Organization) [2] to capitalize on their pre-existing readiness to use the platform.

Tracking WhatsApp chatter may also result in new opportunities for detecting disease outbreaks. The nascent field of digital epidemiology seeks to model how diseases spread by monitoring digital data sources (eg, through Google search data and Twitter posts) [26]. Although the content of private WhatsApp messages is difficult to track, the volume or nature of messages (eg, forwarded messages) may provide information that can be used to support disease surveillance. To this end, further research is needed to explore the predictive utility of message transmission dynamics on WhatsApp.

### Limitations

In reporting these findings, we noted several limitations. First, we opted to study WhatsApp—the most widely used messaging app. At this juncture, it is unclear whether our results generalize to other messaging apps (eg, Facebook Messenger and Telegram).

Second, our recruitment strategy was limited by the nature of the pandemic. Owing to infectious disease protocols and the short time period when the amount of crisis-related communication was high [27], our data collection process was restricted in terms of the recruitment strategy (web-based sampling), sample size (151 participants), and time frame (1 week per participant). Further research is needed to examine whether our findings generalize to the broader population.

Finally, although the experience sampling method captured WhatsApp usage in participants’ naturalistic settings, the method nonetheless required self-reports. By extending our findings, future studies will benefit from having objective metrics of WhatsApp usage.

### Conclusions

In conclusion, we used the experience sampling method to capture COVID-19 chatter on WhatsApp for the first time. In total, we tracked 924 days’ worth of chatter in situ*,* revealing (1) the sheer prevalence of WhatsApp usage, (2) a typology of WhatsApp users, and (3) a link between usage patterns and constant thoughts about the pandemic. These findings have implications for health communication and disease surveillance, bringing the field 1 step closer to characterizing WhatsApp usage and using these data to gain insights on individual and societal concerns.

## Appendix

Multimedia Appendix 1 Correlation matrix of the seven quantitative WhatsApp usage variables.

Multimedia Appendix 2 Model fit indices for latent profile analysis. Lower absolute values indicate better model fit.

Multimedia Appendix 3 Model parameter estimates (group membership). Parameter estimates for the multi-level model of thoughts about COVID-19 (model 1) and the fear of COVID-19 (model 2) as a function of participants’ group memberships.

## Abbreviations

DASS-21: 21-item Depression, Anxiety and Stress Scale
NUS: National University of Singapore

## References

[1] Singh M (2020). WhatsApp hits 2 billion users, up from 1.5 billion 2 years ago. TechCrunch.

[2] Liu JCJ, Tong EMW (2020). The relation between official WhatsApp-distributed COVID-19 news exposure and psychological symptoms: Cross-sectional survey study. J Med Internet Res.

[3] (2020). COVID-19 barometer: Consumer attitudes, media habits and expectations. Kantar.

[4] Newton C (2020). WhatsApp puts new limits on the forwarding of viral messages. The Verge.

[5] Singh M (2020). WhatsApp’s new limit cuts virality of ‘highly forwarded’ messages by 70%. TechCrunch.

[6] Bapaye JA, Bapaye HA (2021). Demographic factors influencing the impact of coronavirus-related misinformation on WhatsApp: Cross-sectional questionnaire study. JMIR Public Health Surveill.

[7] Luk TT, Zhao S, Weng X, Wong JYH, Wu YS, Ho SY, Lam TH, Wang MP (2021). Exposure to health misinformation about COVID-19 and increased tobacco and alcohol use: a population-based survey in Hong Kong. Tob Control.

[8] Lwin MO, Lu J, Sheldenkar A, Schulz PJ, Shin W, Gupta R, Yang Y (2020). Global sentiments surrounding the COVID-19 pandemic on Twitter: Analysis of Twitter trends. JMIR Public Health Surveill.

[9] Chen E, Lerman K, Ferrara E (2020). Tracking social media discourse about the COVID-19 pandemic: Development of a public coronavirus Twitter data set. JMIR Public Health Surveill.

[10] Gireesh KV, Dineshan K (2019). Social media misinformation in disaster situations: A case of 2018 Kerala flood. Library Progress (International).

[11] Simon T, Goldberg A, Leykin D, Adini B (2016). Kidnapping WhatsApp – Rumors during the search and rescue operation of three kidnapped youth. Comput Human Behav.

[12] Lottridge D, Bentley FR (2018). Let's hate together: How people share news in messaging, social, and public networks.

[13] Csikszentmihalyi M, Larson R, Csikszentmihalyi M (2014). Validity and reliability of the experience-sampling method. Flow and the Foundations of Positive Psychology.

[14] Hektner JM, Schmidt JA, Csikszentmihalyi M (2007). Experience Sampling Method: Measuring the Quality of Everyday Life.

[15] Vosoughi S, Roy D, Aral S (2018). The spread of true and false news online. Science.

[16] Lovibond PF, Lovibond SH (1995). The structure of negative emotional states: comparison of the Depression Anxiety Stress Scales (DASS) with the Beck Depression and Anxiety Inventories. Behav Res Ther.

[17] Mesch GS, Schwirian KP (2019). Vaccination hesitancy: fear, trust, and exposure expectancy of an Ebola outbreak. Heliyon.

[18] Scrucca L, Fop M, Murphy TB, Raftery AE (2016). mclust 5: Clustering, classification and density estimation using Gaussian finite mixture models. R J.

[19] Wade TD, Crosby RD, Martin NG (2006). Use of latent profile analysis to identify eating disorder phenotypes in an adult Australian twin cohort. Arch Gen Psychiatry.

[20] Bolger N, Laurenceau JP (2013). Intensive Longitudinal Methods: An Introduction to Diary and Experience Sampling Research.

[21] Therneau TM, Atkinson EJ, Mayo Foundation (1997). An introduction to recursive partitioning using the RPART routines. Mayo Clinic.

[22] Grinberg N, Joseph K, Friedland L, Swire-Thompson B, Lazer D (2019). Fake news on Twitter during the 2016 U.S. presidential election. Science.

[23] Guess A, Nagler J, Tucker J (2019). Less than you think: Prevalence and predictors of fake news dissemination on Facebook. Sci Adv.

[24] Moulds ML, Kandris E, Starr S, Wong ACM (2007). The relationship between rumination, avoidance and depression in a non-clinical sample. Behav Res Ther.

[25] Blabst N, Diefenbach S (2017). WhatsApp and wellbeing: A study on WhatsApp usage, communication quality and stress.

[26] Tarkoma S, Alghnam S, Howell MD (2020). Fighting pandemics with digital epidemiology. EClinicalMedicine.

[27] Nsoesie EO, Cesare N, Müller M, Ozonoff A (2020). COVID-19 misinformation spread in eight countries: Exponential growth modeling study. J Med Internet Res.

