# Supplementary figures and images for: Tracking Private WhatsApp Discourse About COVID-19 in Singapore: Longitudinal Infodemiology Study
Source: J Med Internet Res. 2021 Dec 23;23(12):e34218. doi: 10.2196/34218 (PMC8709420; doi:10.2196/34218)

## Appendix A


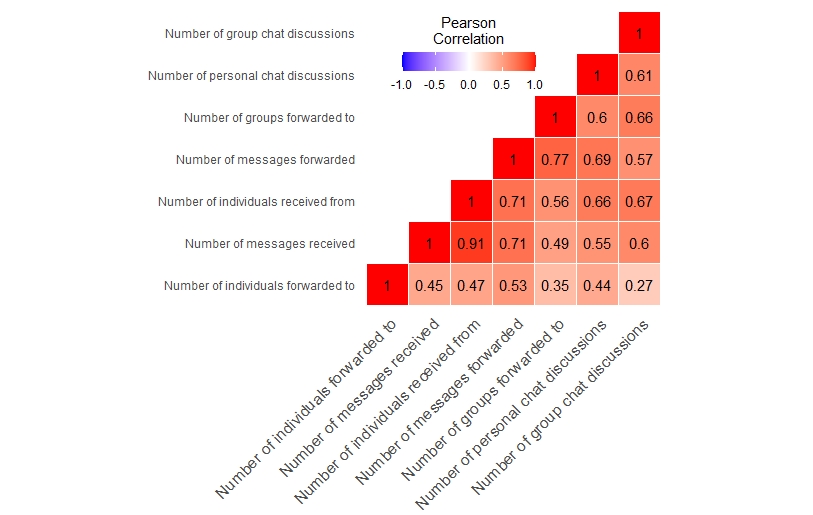


**Figure S1.** Correlation matrix of the 7 quantitative WhatsApp usage variables.

Supplement: Multimedia Appendix 1 [file jmir_v23i12e34218_app1.docx]
